# Person-centered aural rehabilitation program improved mood, cognition, and auditory processing in a professional musician who uses a hearing aid and cochlear implant: Case Report

**DOI:** 10.3389/fresc.2024.1399424

**Published:** 2024-08-07

**Authors:** Christine Brennan, McKenna Spence-Olson, Kayla Cormier, Sherri Tennant, Anu Sharma

**Affiliations:** ^1^ Applied Neuroscience for Communication and Reading, Department of Speech, Language, and Hearing Sciences, University of Colorado Boulder, Boulder, CO, United States; ^2^ Brain Behavior Laboratory, Department of Speech, Language, and Hearing Sciences, University of Colorado Boulder, Boulder, CO, United States; ^3^ Speech, Language, and Hearing Clinic, Department of Speech, Language, and Hearing Sciences, University of Colorado Boulder, Boulder, CO, United States

**Keywords:** person-centered, aural rehabilitation, music, music enjoyment, electrophysiology, case study, hearing loss, home practice program

## Abstract

**Introduction:**

Aural rehabilitation focused on music for individuals with cochlear implants (CIs) and/or hearing aids (HAs) typically emphasizes perceptual skills rather than enjoyment of music. Yet, those with CIs and/or HAs often struggle to enjoy music, complaining that it sounds distorted with the implant or HAs. Typically, aural rehabilitation programs require a significant time commitment, but this may not be feasible or preferable for many patients. This study aimed to evaluate the efficacy of two individualized intensive 3-week home practice programs focused on enjoyment of music, a personal goal for this subject.

**Methods:**

The subject was a professional musician who used a CI and HA. Cognitive measures of global cognitive function, executive function, processing speed, auditory working memory, visual-spatial abilities, verbal fluency, and auditory-verbal memory, as well as auditory electrophysiology (EEG) measures were conducted pre-post experiment 2. Two experiments were undertaken to evaluate responses to two practice programs that incorporated different variations in listening dosage and intervention activities.

**Results:**

Experiment 1 resulted in minimal measurable improvements related to music likability ratings, with the highest dosage condition showing a small increase in average likability rating from baseline to week 3. The results of experiment 2 revealed an improvement in likability ratings only when dosage steadily increased each week. The subject also reported improved mood and decreased frustration during weeks two and three of experiment 2. Finally, we found improvement pre-post experiment 2 on several cognitive and EEG measures.

**Discussion:**

The results of these experiments are encouraging and support the use of an individualized, person-centered, and semi-structured home practice program to increase music enjoyment and improve quality of life and auditory processing for individuals with hearing loss. Future studies should aim to increase sample size and explore pairing person-centered home practice programs with concurrent clinician-lead aural rehabilitation.

## 1 Introduction

Music in daily life is important for social functions, relaxation, and personal enjoyment (1), and it has been found to improve mood, energy, and pain in older adults (2). The presence of hearing loss (HL) is associated with changes to the range of frequencies a person can hear, reducing their access to music (3) and decreasing quality of life. Hearing aids (HAs) and cochlear implants (CIs) improve speech perception but distort and degrade music perception (4–6). Specifically, HAs can distort music due to limitations of the frequency range, irregular frequency response, artifacts produced by feedback-cancellation systems, frequency shifting (if activated), processing time delays, and distortion for high sound levels (7). CIs are associated with a reduced frequency resolution due to broad bandpass filtering, a limited CI frequency input range, and imprecise electrical stimulation of the auditory nerve (8). This may result in music that sounds out of tune, distorted, or strange. As a result, the current state of these technologies does not provide full access to music and perception of music features often requires auditory training (9). For CI users, it may take up to a year to reach a stable map (i.e., optimized CI settings for an individual) and experience improvement in speech perception (10). Establishing a stable map for music perception may take even longer. For HA and CI users, auditory training also improves music perception (9), but patients report that the training schedules are unrealistic (9) and evidence-based interventions for personalized music-related goals for individuals with HL are limited (9, 11). Due to the importance of music in day-to-day experiences and in quality of life, there is a critical need for person-centered auditory rehabilitation interventions that focus on improving the enjoyment of music for individuals with CIs and HAs. Such interventions should accommodate the individual needs, goals, and preferences of the patients being treated.

Perceptual training is optimal when ecologically valid tasks are used, outcomes align with patient goals, and patient values, preferences, and experiences are considered (12, 13). Music interventions for HL typically target perception (e.g., identification of instruments and pitch) rather than enjoyment (9), creating a limitation in the ability of such interventions to align with patient goals, values, and preferences. Interventions that target enjoyment focus on music selection (e.g., selecting music that emphasizes rhythm vs. melody) (14) rather than directly addressing the issues that lead to reduced music enjoyment. By failing to consider individual needs and goals [see (9), for a review], these existing intervention programs fail to improve enjoyment for the music patients prefer.

The schedule and duration of aural rehabilitation sessions that focused on music training after CI implantation impact music perception and enjoyment for those with HL (1, 9, 15–17). Effective interventions that aim to improve perception of music typically require 1 week to several months of therapy (15, 17, 18). Shorter sessions distributed over multiple days may be more manageable (14), which is critical since patients may be unable or unwilling to adhere to long-term, intensive intervention schedules (9). Musical training using excerpts of various instruments resulted in improved recognition of timbre after as few as 3 weeks of perceptual training (15). The 3-week timeframe is notable considering that the typical time needed for improvement in music tasks often spans months and may not be realistic for many people (9, 15). For CI users to have improved perception of musical characteristics, effective intervention appears to require consistent repeated listening (9, 15, 19).

HL also affects neural pathway organization. The cortical auditory evoked potential (CAEP) is an objective measure of auditory cortex plasticity (20). In adults, three obligatory components of the CAEP (P1, N1, and P2) reflect development of the thalamus and the primary and secondary auditory cortices (21, 22). Following amplification, CAEP responses are characterized by changes to the latency and amplitude of these components (23–27). Short-term auditory training increased CAEP N1 and P2 amplitudes (28–30) and decreased CAEP N1 latency (31). The CAEP response appears to be an objective measure that captures neuroplastic changes following listening therapies.

While access to sound helps the neural pathway organize and mature, untreated HL is associated with cognitive decline (32–34) and HA/CI use is associated with better cognitive outcomes (35–39). Daily use of an amplification system for longer durations of time is associated with greater cognitive improvement (37). Older adults who increased the daily time they spent listening to music resulted in improvement on a working memory measure, suggesting that music exposure may impact cognition more directly (40). Therefore, if HA and CI users avoid wearing their devices due to dissatisfaction with sound quality, including music, they may face an increased risk of cognitive decline. Conversely, effective music enjoyment intervention programs can encourage extended device use, potentially improving cognitive outcomes.

Despite the important role music can have on quality of life (1, 2), previous studies have not considered the personal goals, schedule needs, or music preferences of those with HL when creating music enjoyment interventions (9). To address this limitation of past research, this single case study provided two practice programs and systematically evaluated their effects on music enjoyment, cognition, and auditory processing.

## 2 Case description

**Figure 1:**
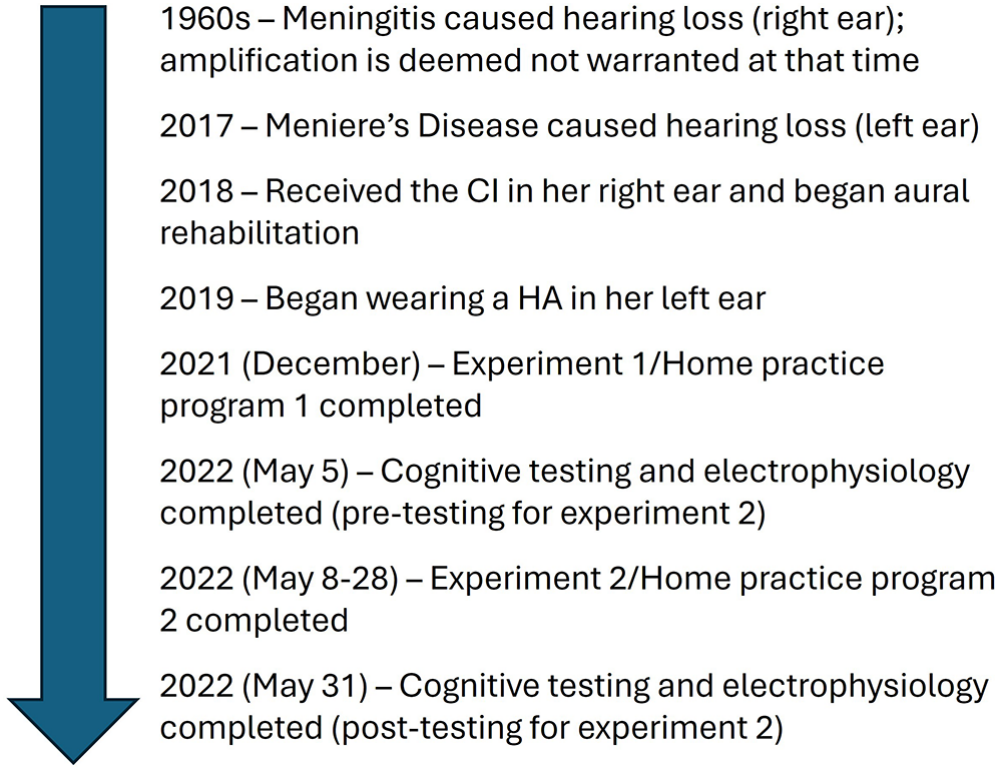
Timeline.

The subject was a 63-year-old retired professional chamber musician with bilateral hearing loss who used a Phonak Marvel Audeo M-90-RT HA (left ear) and a Med-El CI (right ear) (see Figure 1 for the subject's timeline). She lost hearing in her right ear during childhood after contracting meningitis but did not require amplification at that time. In 2017, the subject was diagnosed with Meniere's Disease, which significantly reduced her hearing in her left ear. In 2018, she received the CI in her right ear and began wearing a HA in her left ear in 2019. In 2021, she reported difficulty tolerating the CI for long periods of time, especially in noisy environments. At that time, she was working with an audiologist, progressing through a series of listening programs for her CI with each program increasing in volume. Once she felt the amplification settings for the CI were tolerable, she sought additional auditory training with a specific interest in music. At the start of experiment 1, she reported wearing her CI and HA for all waking hours.

The subject reported that she typically studied, played, and listened to music daily, but after receiving her CI no longer did these activities due to music distortion. Prior to this study, she received aural rehabilitation focused on speech discrimination and comprehension and perception of music features for a limited number of instruments. She expressed interest in improving her enjoyment of music and expanding the number of instruments she could enjoy.

Review of this study was waived by the institutional review board (IRB) at the University of Colorado Boulder. This IRB does not review single case studies and advised the research team to take steps to protect subject privacy and provide informed consent. Informed written consent was obtained before all aspects of this study. No identifying information is reported here to protect subject privacy.

### 2.1 Diagnostic assessment methods

Cognitive testing and EEG (CAEP) were completed before and after the second home practice program (experiment 2). Due to safety concerns from the COVID-19 pandemic, these measures could not be obtained before and after the first at home practice program (experiment 1).

#### 2.1.1 Cognitive assessment

A cognitive test battery was completed prior to the start of experiment 2 and repeated 30 days after experiment 2 was completed. The cognitive test battery followed the protocol described in Glick and Sharma (35) and included the following measures: MoCA (The Montreal Cognitive Assessment) was used to assess global cognitive function (41); BDS-II (Behavioral Dyscontrol Scale II) was used to assess executive function (42); SDMT (Symbol Digits Modalities Test) was used to assess processing speed (43); WAIS-IV Digits Backward Subtest was used to assess auditory working memory (44, 45); WAIS-IV Block Design Subtest was used to assess visual-spatial abilities (44, 45); COWALT (Controlled Oral Word Association Test) was used to assess verbal fluency (46); and the RAVLT (Rey Auditory Verbal Learning Test) was used to assess auditory-verbal memory (47).

#### 2.1.2 Electrophysiological measures

CAEP responses were measured to examine neuroplastic changes in the auditory cortex using the methodology described in Campbell and Sharma (48). CAEP measurements were collected pre and post experiment 2, on the same day as the cognitive testing. Sound field measurements, at 45° azimuth delivered by two loudspeakers, were obtained at each time point A synthesized speech syllable/ba/ with a duration of 90 ms at an intensity of 60 dB HL was utilized to elicit the cortical auditory evoked response (22, 49, 50). Nine electrodes were utilized to obtain CAEP responses in order to minimize any artifacts arising from the CI (51). The active electrode was located at Cz, the ground electrode located at Fpz, and the remaining seven electrodes were positioned along the isopotenital contour. Eye blinks were monitored with electrodes placed at the superior and lateral canthus. All testing took place in an electromagnetically shielded sound booth with the subject seated in a comfortable chair. Ear specific information was obtained by testing the subject's HA and CI separately. Given the subject's degree of hearing loss, they were unable to hear the stimulus in the contralateral ear without the use of a device. The subject's CI and HA were set to their typical settings for all testing.

The cortical responses were recorded on a Compumedics Neuroscan system and analyzed using the Scan acquisition software. A sampling rate of 1,000 Hz was employed, and the data were filtered from 0.1 to 1,000 Hz. At least two runs of each condition at each time point were completed to ensure the replicability of responses. Within each run at least 250 epochs, including a 100 ms pre-stimulus and 600 ms post-stimulus time window, were obtained. Epochs containing movement artifacts, identified using a cutoff of ±100 µV, were rejected.

### 2.2 Data collection procedures

#### 2.2.1 Person-centered home practice program 1 (experiment 1)

Instructions and data sheets for the first 3-week daily home practice program were given to the subject during one of her final aural rehabilitation sessions (see Supplementary Material A).

**Figure 2:**
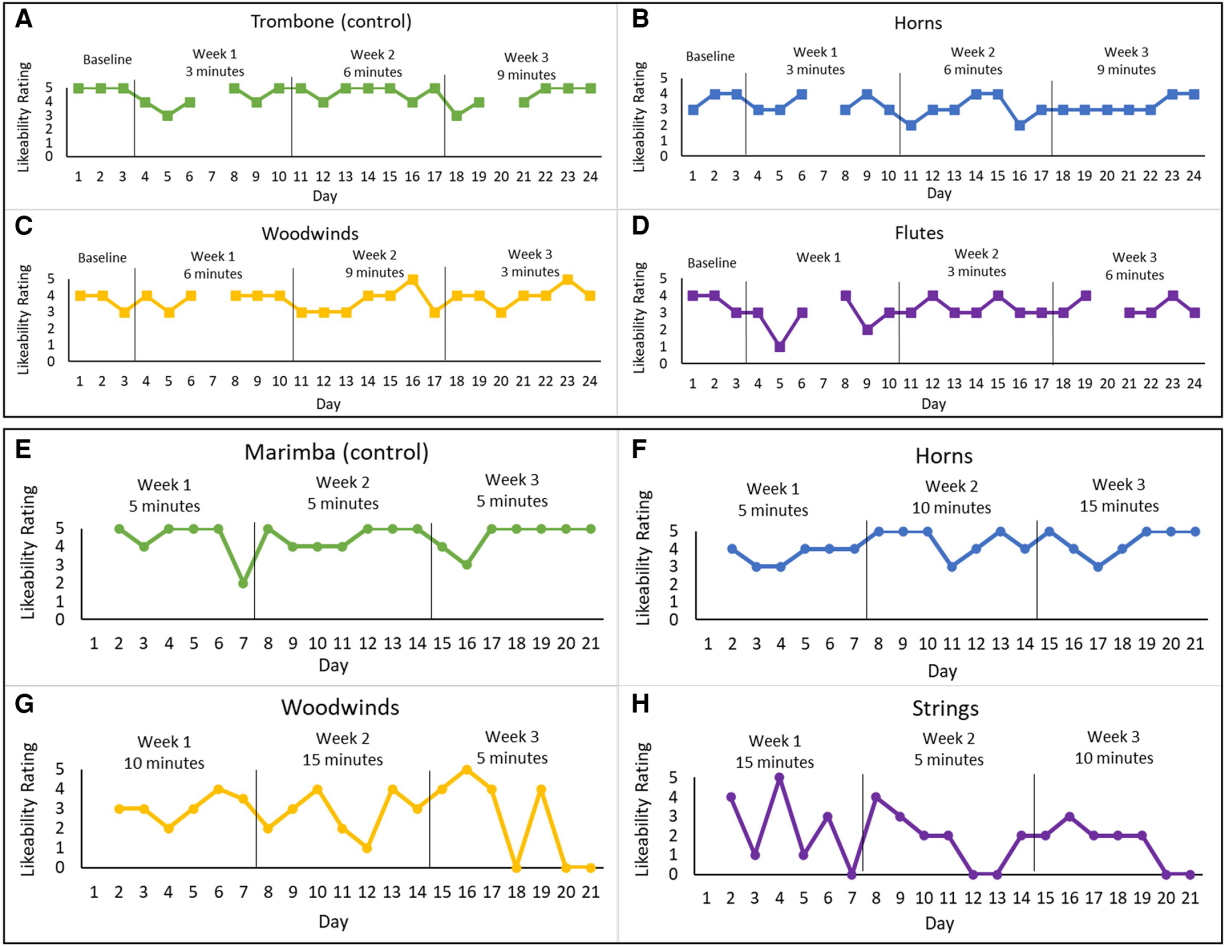
Likeability ratings for both home practice programs for the different instrument-dosage conditions. Experiment 1: (**A**) trombone (control)—3, 6, and 9 min, (**B**) horns—3, 6, and 9 min, (**C**) woodwinds—6, 9, and 3 min, and (**D**) flutes—9, 3, and 6 min. Experiment 2: (**E**) Marimba (control)—5 min each week, (**H**) strings—15, 5, and 10 min, (**G**) woods—10, 15, and 5 min, and (**F**) horns—5, 10, and 15 min. The graphs present the daily likeability ratings for each instrument group across the 3-week home practice programs.

The home practice program was completed by the subject in the bimodal mode (CI + HA) independently at home. The program included three listening dosage conditions (daily listening times) and one control condition. The dosage changed for each instrumental group each week (3, 6, or 9 min) for four different instrument groups (horns, flutes, other woodwinds, and trombones). The control condition (trombone) had high likeability ratings prior to the intervention. Each week assigned dosages were changed and maintained for the week (see Figures 2A–D). Previous studies included listening dosages of between 10 and 30 min (16, 18). Therefore, we selected a total listening dosage for each week that fell within this range (21–27 min) for the first experiment.

The subject listened to music recordings daily for each condition for the prescribed listening dosages. Total daily listening minutes aligned with a previous listening therapy study (16). During each home session, the subject rated the likability of each instrument (dosage condition). The likeability scale was used by the subject in her previous aural rehabilitation sessions and used a Likert scale from 1 to 5 (1 being not enjoyable and 5 being highly enjoyable).

The subject chose recordings from a clinician created list of online music links that matched this subject's listening assignments and preferences (e.g., no accompanying instrumentation) and had minimal background noise. She received no other aural rehabilitation while completing the home practice program.

#### 2.2.2 Person-centered home practice program 2 (experiment 2)

The second home practice program was completed several months after completing the first home practice program. Several changes were implemented for the second program that aimed to improve upon the initial program and to accommodate specific feedback and requests made by the subject. The procedures followed those from the first program (see Supplementary Material B for the instructions given to the subject). Per subject request, the listening dosages were increased to 5, 10, and 15 min (see Figures 2E–H). These dosages resulted in a consistent total daily listening dosage of 35 min (compared to the varied daily dosages of 21–27 min in Experiment 1). The increased dosage was just above the 30-minute dosage used by Galvin et al. (16) and was within the total time that the subject reported as feasible given her daily schedule and comfort with listening to music. The subject also expressed interest in listening to marimba, which she recently realized she enjoyed, and requested that this be included in the second home practice program. This request was accommodated by making marimba the control for the second program. The number of instruments in each recording also increased. Several other additions included guided listening questions, daily pre- and post-program questions about mood and self-confidence, and a weekly music enjoyment survey (see Supplementary Material C). The likeability scale and the guided listening questions used here aligned with what this subject was familiar with and had used in her clinical program prior to starting this study. The daily questions about mood and self-confidence and the weekly questions about music enjoyment were created by the research team based on feedback this subject provided after completing program 1. Since her previous feedback included some information about her mood and confidence, questions were generated specifically to gather this information following program 2. During the time she completed the second home practice program, the subject did not participate in any other aural rehabilitation.

The instruments included horns, woodwinds, strings, and marimba (control). A new list of recordings with YouTube links was provided. Most of the recordings were new, but some were also used in the first program.

## 3 Results

### 3.1 Results following home practice program 1

The subject missed some assignments but was generally compliant and completed the program. There was a slight improvement in the average likability ratings for one condition (woodwinds) with weekly dosages of 6, 9, and 3 min (see Figure 2C). The subject reported that she enjoyed the program and believed her enjoyment of music had increased, even though this was not strongly reflected in her likeability ratings across conditions.

The subject reported several positive qualitative changes following the home practice program, specifically, more easily recognizing music and able to listen to instruments previously avoided.

It should be noted that during this first home practice program, there were unexpected CI listening program changes following an appointment with the subject's audiologist. These changes included adjustments to volume levels for select frequencies. These changes may or not have had an impact on likeability (positive or negative). Additionally, the subject did have some missed listening assignments that could have minimized improvements in likeability outcomes.

### 3.2 Results following home practice program 2

#### 3.2.1 Likeability and survey responses

Due to personal circumstance, the subject did not complete a baseline and skipped the first day of week 1 so that she could complete the program before a planned vacation. The likeability rating results of experiment 2 showed an increase in likeability for one of the experimental conditions (horns) (see Figure 2F). The control condition also showed a slight increase in likeability rating (see Figure 2E).

A thematic analysis was done for the daily pre- and post-program questions about mood and self-confidence and the weekly music enjoyment surveys that were completed during the second experiment. The approach for this analysis followed a simplified version of that described by Braun & Clarke (52). This included reviewing the subject responses for each day/week and searching for themes. The subject's responses to the daily question about mood were single words and included content, relaxed, tired, irritated, and frustrated, and ok. Given these responses to the mood questions, we identified three themes, specifically positive, negative, or neutral emotions. For questions about mood, positive responses included content and relaxed, negative responses included tired, irritated, and frustrated, and neutral responses including ok. A review of the themes coded revealed that the subject's responses to the mood questions indicated she was feeling tired, frustrated, and irritated after 4 out of 7 days during week 1, but had fewer instances of fatigue during week 2. During the third week, she reported positive feelings (e.g., relaxed or content) most days. The subject also reported high or moderate self-confidence before listening and moderate after listening. The subject's responses to the post-program survey revealed the importance of the structured music listening routine. Specifically, the subject stated that the program “created a music listening routine I would not have followed otherwise. Continuing a program similar to this and with quality improvement with fine tuning mappings will hopefully bring back music enjoyment. This is a first step and very valuable.”

### 3.3 Cognitive measures

Table 1 displays baseline, post-intervention, and difference scores on all cognitive measures. There was an 8-point increase on the Rey Auditory Verbal Learning Test (RAVLT) and a 31% increase in the percent of target words recalled after a 20-min delay following intervention. There was also a 16-word increase in repeated interference words on the RAVLT. There was a 12-point increase in phonemic verbal fluency on the Controlled Oral Word Association Test (COWAT). In contrast, there was a 17-point decrease in visual-spatial abilities (Block Design). Other cognitive measures demonstrated negligible changes.

**Table 1:** Cognitive test scores pre-and post-intervention and the change in cognitive test scores.

Cognitive test	Intervention status		
	Pre	Post	Change
RAVLT percent recall	69.23	100	30.77
RAVLT intrusions	67	83	16
RAVLT total recall	45	53	8
COWAT	44	56	12
SDMT	58	60	2
MoCA global score	27	26	−1
Digit span backwards	9	8	−1
BDS-II	26	24	−2
Block design	64	47	−17

MoCA, the Montreal cognitive assessment was used to assess global cognitive function (41); BDS-II, behavioral dyscontrol scale II was used to assess executive function (42); SDMT, symbol digits modalities test was used to assess processing speed (43); WAIS-IV digits backward subtest was used to assess auditory working memory (44, 45); WAIS-IV block design subtest was uses to assess visual-spatial abilities (44, 45); COWALT, controlled oral word association test was used to assess verbal fluency (46); RAVLT, Rey auditory verbal learning test was used to assess auditory-verbal memory (47).

### 3.4 Electrophysiological measures

**Figure 3:**
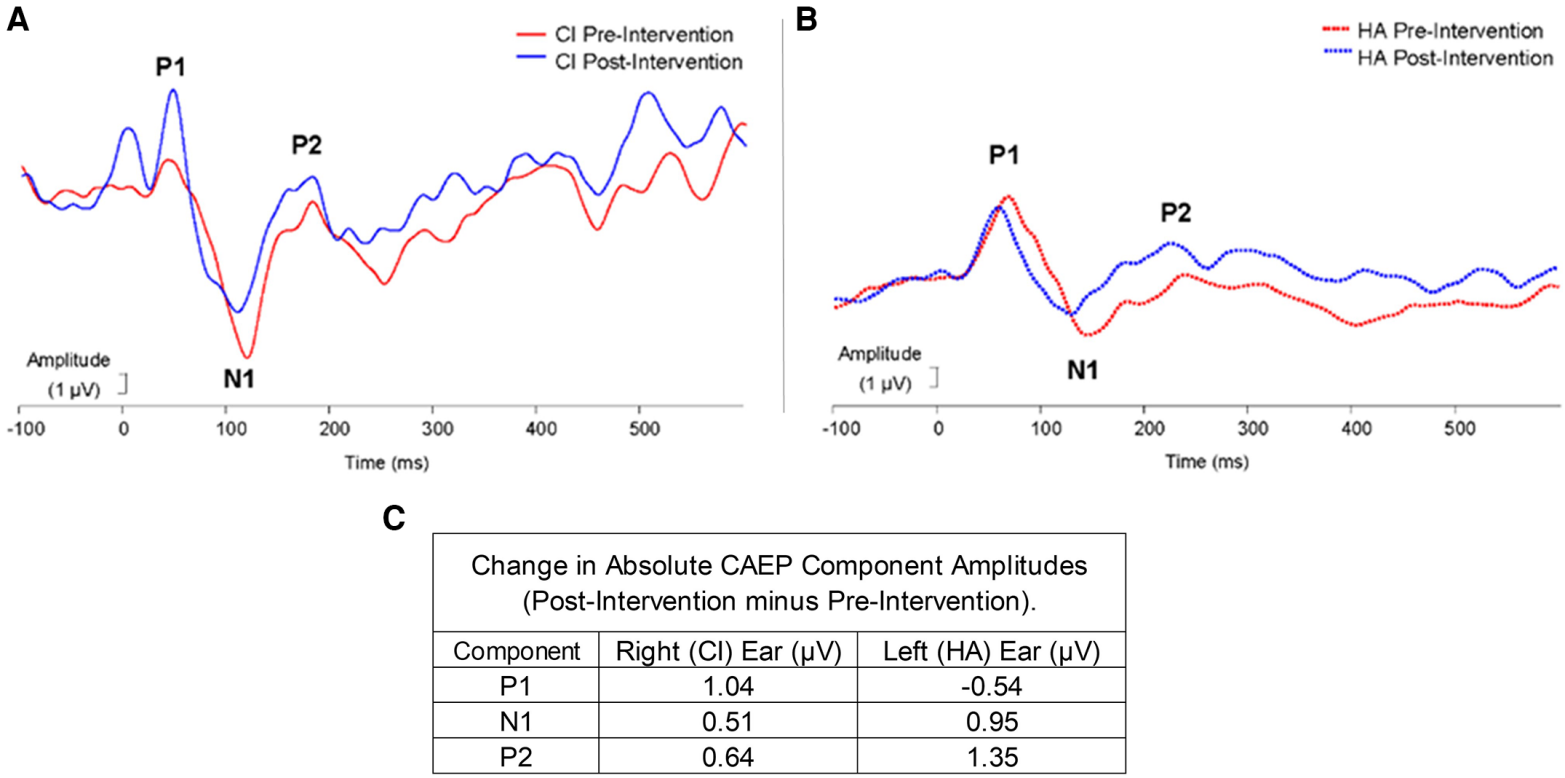
Experiment 2: pre-post changes in CAEP responses for CI (**A**) and HA (**B**) time (ms) is plotted on the x-axis and amplitude (µV) is plotted on the y-axis. Peaks P1, N1, and P2 are labeled in each graph. The red lines represent pre-intervention amplitude, and the blue lines represent post-intervention amplitude. (**C**) Shows the amplitude change (µV) from the CAEP responses for the Right (CI) and Left (HA) ears.

All components of the CAEP response (P1, N1, P2) were observed for each ear for this subject. For experiment 2, the N1 latencies changed pre- to post-intervention in both ears (see Figure 3). Specifically, in the right ear (CI) following the intervention, the N1 latency decreased by 11.02 ms from the pre-intervention latency. The P1 and P2 components in the CI ear demonstrated negligible latency changes. Similar latency shifts in the N1 component were noted in the left ear (HA). The decrease in latency from pre-intervention to post-intervention N1 responses in the HA ear was 14.02 ms. Moreover, the HA ear showed a decrease in latency for the P2 component from pre- to post- intervention of 16.02 ms. However, the P1 latencies across time points were comparable in the HA ear.

Absolute changes in amplitudes for each component in each ear are exhibited in Figure 3C. In the right ear (CI), the largest amplitude increase occurred in the P1 component (1.04 µV), while in the left ear (HA) the largest amplitude increase was noted for the P2 component (1.35 µV). P1 to N1 peak-to-peak amplitude increased by 0.52 µV following intervention and the N1 to P2 peak-to-peak amplitude increased by 0.13 µV in the right ear. In the left ear, the P1 to N1 peak-to-peak amplitude decreased following the intervention by 1.49 µV and the N1 to P2 peak-to-peak amplitude increased following the intervention by 0.40 µV.

## 4 Discussion

Survey responses indicated that the subject believed her enjoyment of music had increased following the first home practice program, but improvement in weekly average likeability was only found in one condition (i.e., woodwinds) and that improvement was marginal. Given the small increase in likability, it is possible that this dosage pattern (6, 9, 3) was beneficial for this subject. It is possible that improvements in likeability require initial dosages of at least 6 min per day but given the limited data here this is speculative.

Weekly average likeability also increased for one condition in the second program (i.e., horns). In this experiment, improved likeability was associated with dosages that increased each week (5-, 10-, and 15-min). No other instrument group in this second program had weekly increases in dosage. This may suggest that increased dosages over time are more beneficial to music enjoyment than decreased or variable dosages. Taken together, the findings from both experiments may suggest that initial listening dosage should be at least 5–6 min and that dosage should increase each week.

The subject reported enjoying the second program and found it beneficial even though likeability ratings did not change appreciatively across conditions. It is possible that the likeability scale was not sensitive enough to detect meaningful changes, resulting in a discrepancy between ratings and perception of improvement. It is also possible that there were changes in enjoyment that were not specific to the instrument groups or dosages, but instead reflect general changes in listening that were not measured. It is unknown if the positive qualitative changes reported by the subject were due to changes in auditory processing or to other factors, such as improvements in confidence, mood, and/or attitude.

The thematic analysis of the mood and confidence survey revealed three themes, positive negative, and neutral motions. The subject responses indicated that irritation and frustration had decreased over the 3 weeks of the second program, with more positive responses and no negative responses about mood during week 3. The improvement in mood after listening assignments in the second program is notable since this second program included more music that was less preferred by this subject (i.e., ensembles and strings). This improvement may be due to the increased dosages (per instrument group and total daily listening time), better program compliance, and/or the inclusion of guided listening questions.

The post-program survey indicated that the subject valued the structured listening routine, suggesting that this structured home-based listening program was beneficial as part of her aural rehabilitation program.

### 4.1 Cognitive changes

Post-experiment 2 increases were noted in verbal fluency and auditory-verbal memory, consistent with previous studies that reported increases in cognitive abilities following the adoption of HA/CIs (35–38, 53). Previously, cognitive improvements occurred primarily after the first 6 months or year of device use, but the current subject had been wearing her CI and HA for several years. We suggest that the cognitive changes found here are the result of the second home practice program. It is important to note that although cognitive changes may have occurred with experiment 1, cognitive changes were only documented pre-post-experiment 2.

The current subject showed a decrease in visual-spatial abilities. Previous studies reported increased visual attention in postlingually deafened adults compared with normal hearing peers (54–57). The decrease in visual-spatial processing noted here may reflect improved auditory processing and a decreased focus on visual processing.

### 4.2 Electrophysiological changes

Similar to previous research on CAEP changes with auditory training, we observed objective neuroplastic changes in the central auditory pathways, including latency changes in the later N1 and P2 components, rather than the earlier P1 component (28–31). Latency decreases in the N1 CAEP were the most obvious, consistent with previous findings of a decrease in N1 latencies following a 2-week listening therapy with degraded music stimuli (31). Significant decreases in N1 latency were also found in post-lingually deafened adults following the first 8 weeks of CI usage (58). Additional research is needed to examine the effect of device use and listening therapies separately.

We also observed an increased P1 and P2 amplitudes in the CI and HA ears respectively. Previous investigations of speech listening training also showed significant changes in CAEP amplitudes (28–30). Latency decreases, and amplitude increases reflect improvements in efficiency of neural auditory pathways, suggesting that the second experiment successfully targeted refinements in central auditory processing. It is important to note that although electrophysiological changes may have occurred with experiment 1, changes were only documented pre-post-experiment 2.

### 4.3 Clinical implications

Given the importance of exposure to music post-implantation for auditory rehabilitation of music (1), it is encouraging that the increased listening dosage of the second home practice program was still feasible for this subject. While further study is still warranted, the results of the current study suggest that other adults with hearing loss who seek to improve enjoyment of music may benefit from a structured and individualized home practice program. Since we did not specifically measure quality of life, we cannot definitively state that there is improvement, but in this case, the subject's responses to the post-program survey questions provide preliminary evidence that the person-centered programs implemented here improved mood and decreased frustration. The use of a person-centered approach in this study provides a model for how an aural rehabilitation program targeting music enjoyment could be individualized based on individual patient needs, goals, and preferences.

### 4.4 Limitations and future research

All case studies have inherent limitations and the results found here may not generalize to others. A notable limitation here is the subjectivity of the unvalidated likeability ratings and the possibility that these ratings were not able to capture change in music enjoyment. The use of unvalidated measures of likeability makes it difficult to interpret the ratings with confidence. The surveys were also subjective and may not be as reliable as the objective measures. To compensate for these subjective measures, the use of cognitive assessments and electrophysiology provided objective measures with little to no subjectivity.

The reported case is a musician who was most likely well-trained in the perception of music prior to adoption of the HA/CI. Given her previous musical training and her high motivation to improve music enjoyment, she may have been more compliant than a non-musician. Although the subject had prior musical experience, the results of this case report are encouraging and suggest the need for further research. Future studies should focus on enhancing music enjoyment for non-musicians who use hearing aids and/or cochlear implants.

Future investigations should consider using more individualized and customized protocols created for music and auditory training. Future investigations should evaluate home practice programs paired with weekly auditory rehabilitation sessions lead by a clinician, as recommended by Looi et al. (9). The 3-week period for the subject in this study was important due to her schedule. Therefore, future studies should aim to explore the efficacy of person-centered, intensive, and semi-structured home practice programs longer than 3 weeks as well as programs that do or do not require daily listening. In this study, ratings for mood and self-confidence may reflect improvement and changes in quality of life more than the likability scale. Future researchers may consider investigating other ways of measuring music enjoyment that more accurately capture and reflect improvement.

### 4.6 Conclusion

This aural rehabilitation case study involved two 3-week home practice programs focused on music enjoyment for a musician who used a CI and HA. Small changes in likability were associated with progressively increased listening dosages. Following the second program, the subject reported decreased irritation and frustration and positive changes in cognitive scores. Electrophysiological findings support positive changes in cortical pathways following intervention. These results collectively reveal that the two individualized home practice programs effectively changed this subject's perception that her enjoyment of music was improving, as well as her mood, cognitive skills, and auditory processing. The current results are encouraging and support future use and investigation of person-centered home practice programs to improve music enjoyment for individuals with HL. Future studies should increase sample size and explore pairing individualized home practice that consider patient needs, goals, and preferences with concurrent clinician-lead, structured, personalized aural rehabilitation.

## Data Availability

The raw data supporting the conclusions of this article will be made available by the authors, without undue reservation.
