# Supplementary material for: Person-centered aural rehabilitation program improved mood, cognition, and auditory processing in a professional musician who uses a hearing aid and cochlear implant: Case Report
Source: Front Rehabil Sci. 2024 Aug 7;5:1399424. doi: 10.3389/fresc.2024.1399424 (PMC11335732; doi:10.3389/fresc.2024.1399424)
Supplement: Supplementary file 1 [file Datasheet1.docx]

Supplementary Material

**Supplementary Material A**

**Instruction Sheet for Experiment 1**

**Timeline**

This program has a baseline period of 3 days followed by 3 weeks (21 days) of home practice listening assignments. A timeline can be found below.

Baseline**: 12/9/2021 to 12/11/2021

Week 1: 12/12/2021 to 12/18/2021

Week 2: 12/19/2021 to 12/25/2021

Week 3: 12/26/2021 to 1/1/2022

**Baseline - The baseline portion of the program provides information about enjoyment of music before the home practice program. You will see that only 1 minute is assigned for each group during the 3 baseline days. For these 3 days, pick a recording for each instrument group and listen to ONLY 1 minute of each recording, then note your likability Score in the appropriate column.

**Data Form Instructions**

On the data form, you will see each day of the program has 4 rows. Each row represents one listening assignment. For each day, please fill out the date and the program you are listening with. Next, note the listening minutes for the first assignment, then navigate to the recordings found on Page 4 of these instructions and select a recording. Listen to the recording for the assigned number of minutes and then rate the recording on the 1-5 likability Scale below. You may listen to multiple recordings or rotate segments of recordings for variability, but please only listen to the assigned number of minutes. Please also note the number associated with the recording in the “Instrument/Song” column. You can leave any additional comments in the Comments column. An example is provided below.

*Likability Scale-*

1. *Not enjoyable at all*
2. *Minimally enjoyable*
3. *Somewhat enjoyable*
4. *Enjoyable*
5. *Very Enjoyable*

**Example for Daily Completion of Assignments**

1. The table below shows the listening assignments for Day 1. To complete the assignments, you would start by writing the date. Also, note which program you are using to listen.


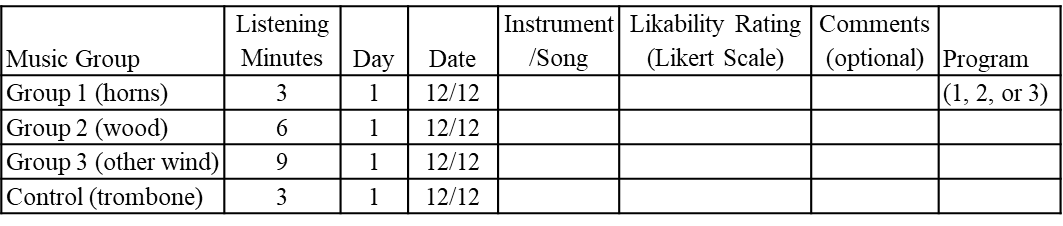


1. Next, you will complete your first assignment, which is to listen to a horn recording for 3 minutes. You will navigate to the instrument recordings page on Page 4 of this document and select a recording from the list. For this example, assume you decide to listen to 3 minutes of the tuba recording. You will note your choice in the “Instrument/Song” column.
2. Next, listen to the recording and then assign the recording a likability score (1-5) using the scale above. Note the score in the appropriate column as shown below. For this example, assume you assign the recording a 3.
3. You can now add any additional comments in the “Comments” column, otherwise you can move on to the next assignment (in this case, the next assignment is listening to wood instruments for 6 minutes).
4. If you decide to use multiple recordings for one assignment, please note all recordings used. For example, the second assignment is to listen to wood instruments for 6 minutes. If you decide to use the first clarinet recording and second bassoon recording for this assignment, you would fill out the table as shown below.

**Missed Days**

Because of the short time frame of this program, missed days are not ideal, however if you need to miss a day, make a note in the comments section of the data sheet and pick up the next day at your current place. Make sure you write the correct date in the “Date” column.

For example, if you completed the assignments for 12/12 but had to miss 12/13, the “Date” and “Comments” columns of your data sheet would look like the one below. As shown, 12/14 would then become Day 2 and you would continue the rest of the program from there.

**Contact Information**

If you have any questions, please reach out to your clinician. Thank you for participating in this home practice program!

**Supplementary Material B**

**Instruction Sheet for Experiment 2**

**Timeline**

This program has a baseline period of 3 days followed by 3 weeks (21 days) of home practice listening assignments. A timeline can be found below.

Baseline**: 3 days

Week 1: 7 days

Week 2: 7 days

Week 3: 7 days

****Baseline -** The baseline portion of the program provides information about enjoyment of music before the home practice program. You will see that 1 minute is assigned for each group during the 3 baseline days. For these 3 days, pick a recording for each instrument group and listen to ONLY 1 minute of each recording, then note your Likability Score in the appropriate column.

**Data Form Instructions**

In the data collection packet you will find one page for each day of the program. For each day, you will note the date then begin answering questions.

First, you will answer the pre-practice questions about your mood and confidence using the empty space and choices following the questions.

Next, you will complete your listening assignments for that day. You will see each day of the program has 4 rows. Each row represents one listening assignment. For each day, please note the CI program you are listening with. Next, note the listening minutes for the first assignment, then navigate to the recordings found on Page 4 of these instructions and select a recording/recordings. Listen to the recording for the assigned number of minutes and then rate the recording on the 1-5 Likability Scale below. You may listen to multiple recordings or rotate segments of recordings for variability, but please only listen to the assigned number of minutes. Please also note the number associated with the recording in the “Song Number” column. You can leave any additional comments in the Comments column. An example is provided below.

*Likability Scale-*

1. *Not enjoyable at all*
2. *Minimally enjoyable*
3. *Somewhat enjoyable*
4. *Enjoyable*
5. *Very Enjoyable*

After completing the listening assignment, answer the post-practice questions, which are the same as the pre-practice questions.

On the last day of each week, you will also answer 4 yes/no questions that can be found on the data sheets for days 7, 14, and 21. You will also have a question asking you to note any speech CI-only practice completed that week.

On the last day of the program, day 21, there are 4 additional open-ended questions.

**Daily Completion of Assignments**

1. Note the date on the top of the data sheet.
2. Answer the pre-practice questions by writing your answer to the first question and circling a choice for the second and third questions.
3. Complete the listening assignments for that day. The table below shows the listening assignments for Day 1. To complete the assignments, listen to a recording for the assigned minutes and note your likability rating. REMEMBER, YOU CAN LISTEN TO MORE THAN ONE RECORDING OR LISTEN TO DIFFERENT PARTS OF A RECORDING TO COMPLETE AN ASSIGNMENT. Also, note which program you are using to listen.


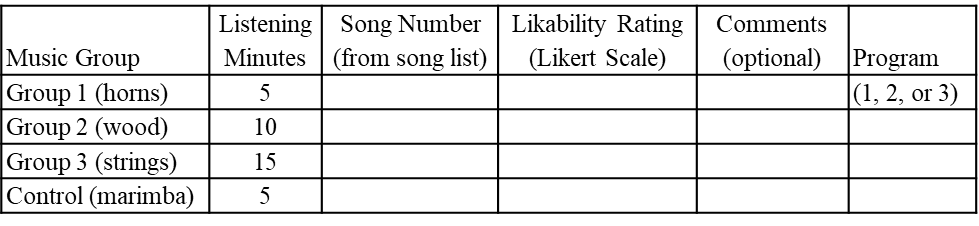


1. First, you will listen to horns for 5 minutes. You will navigate to the instrument recordings page on pages 5-6 of this document and select a recording from the list. For this example, assume you decide to listen to 5 minutes of the second trombone recording (Trombone ii, which is 5 minutes long). You will note your choice in the “Song Number” column.
2. After listening, note your likability rating.
3. You can now add any additional comments in the “Comments” column, otherwise you can move on to the next assignment (in this case, the next assignment is listening to wood instruments for 10 minutes).
4. If you decide to use multiple recordings for one assignment, please note all recordings used. For example, the second assignment is to listen to wood instruments for 10 minutes. If you decide to use Bassoon i and ii and Clarinet i, note all recordings in the data sheet as seen below. Then note your rating of the instrument group (winds) as a whole.

**Missed Days**

Because of the short time frame of this program, missed days are not ideal, however if you need to miss a day, make a note in the comments section of the data sheet and pick up the next day at your current place. **DO NOT CROSS OUT MISSED DAYS.** Rather, continue filling out the sheet as usual. At the end of the program, every data sheet should have data.

For example, if 5/1 is Day 1 and 5/2 is Day 2, say you completed the assignments for 5/1 but had to miss 5/2. On the data sheet for Day 2, write 5/3 as the date and note that 5/2 was missed in the comments section. Then fill out the Day 2 data sheet. ***Following this procedure, you should have no empty data sheets at the end of the program even if there are missed days.***

**Contact Information**

If you have any questions, please reach out to your clinician via email. Thank you for participating in this home practice program!

**Supplementary Material C**

**Guided Listening Questions for Experiment 2:**

1. Is this piece generally loud or generally soft?
2. Do you hear mainly high notes or mainly low notes in this piece?
3. Do you hear any scales in this piece?
4. Are the dynamics relatively stable or do they frequently or suddenly change?
5. When listening to the melody, do you hear mainly ascending, descending, or same notes?
6. If you listened to a duet, which instrument was more pronounced?

Daily Questions about mood and self-confidence:

1. Describe your mood right now.
2. Can you rate how strong your mood is right now? (Choices: Extremely, Moderately, Mildly, Weakly)
3. Rate your level of general self-confidence right now. (Choices: Very High, High, Moderate, Low, Very Low)

Weekly questions about music enjoyment (asked at the end of each week):

*(choices were: yes, no, I don’t know, and prefer not to answer)*:

1. Do you enjoy listening to music more after completing the assignments this week?
2. Did you notice more characteristics of the music for one or more instruments this week in comparison to previous weeks? (For week 1, did you notice more characteristics of the music this week than the last time you listened to music before starting the program?)
3. Have you noticed changes to the music you hear outside of the program since beginning this program?
4. Do you feel that you enjoyed one instrument group more than the others this week? (If yes, please note which group)

Post-intervention survey (asked after the program was completed):

1. You have mentioned that music is an important part of your quality of life, do you feel like this program has added to your quality of life by targeting music enjoyment?

2. If you were going to complete this program a third time, or if this program would be recommended for other people, what would you change?

3. What is your general opinion of this program? What did you like most about it?

4. Do you think that your enjoyment of listening to music outside of the program has changed? Can you describe this change?

**Supplementary Material D**

**Mood and self-confidence ratings pre-practice and post-practice by day.**

| **Day** | *Describe your mood right now?* | | *Can you rate how strong your mood is right now?* | | *Rate your level of general self-confidence right now.* | |
| --- | --- | --- | --- | --- | --- | --- |
|  | ***Pre*** | ***Post*** | ***Pre*** | ***Post*** | ***Pre*** | ***Post*** |
| **1*** | - | - | - | - | - | - |
| **2** | Relaxed | Content; relaxed | Moderately | Moderately | High | High |
| **3** | Tired | More Tired | Moderately | Moderately | no response | Low |
| **4** | Relaxed | Content | Moderately | Moderately | High | Moderate |
| **5** | Relaxed | Irritated after violin; content after marimba | no response | Moderately | no response | Moderate |
| **6** | Relaxed | Irritated after cello; relaxed after marimba | Moderately | no response | High | no response |
| **7** | Relaxed | Frustrated; irritated | Moderately | Moderately | High | Moderate |
| **8** | Relaxed | Relaxed | Moderately | Moderately | High | High |
| **9** | Content | Content | Moderately | Moderately | Moderate | Moderate |
| **10** | Relaxed | Relaxed | Moderately | Moderately | High | High |
| **11** | Tired | Ok | Extremely | Moderately | Moderate | Moderate |
| **12** | Tired | Tired | Moderately | Moderately | Moderate | Moderate |
| **13** | Tired | Ok | Moderately | Moderately | Moderate | Moderate |
| **14** | Happy | Relaxed | Moderately | Moderately | High | High |
| **15** | Relaxed | Content | Moderately | Moderately | High | Moderate |
| **16** | Relaxed | Ok | Moderately | Moderately | High | Moderate |
| **17** | Content | Content | Moderately | Moderately | High | Moderate |
| **18** | Content | Content | no response | Moderately | no response | Moderate |
| **19** | Content | Ok/tired | Moderately | Moderately | Moderate | Moderate |
| **20** | Relaxed | Content | Moderately | no response | High | Moderate |
| **21** | Relaxed | Ok | Moderately | Moderately | High | Moderate |

*The subject reported that skipping Day purposely so that she could complete the program before leaving for a vacation trip.
